# Physiological Ecology and Regulation of High-Yield Maize Cultivation

**DOI:** 10.3390/plants15172569

**Published:** 2026-08-24

**Authors:** Wanrong Gu, Jing Li

**Affiliations:** College of Agronomy, Northeast Agricultural University, Harbin 150030, China

Maize (*Zea mays* L.) stands as the world’s most productive cereal crop, underpinning food security, feed supply, and industrial raw material provision across temperate and continental agroecological zones. Global demand for maize grain continues to surge amid shrinking arable land, erratic climate stress, and rising constraints on nitrogen input and greenhouse gas emissions. To reconcile high yield potential with lodging resistance, resource-use efficiency, and environmental sustainability, modern maize agronomy has shifted from single-factor management toward integrated regulation of population structure, nitrogen fertilization, plant growth regulators (PGRs), soil conservation practices, intercropping configurations, and stress mitigation biostimulants. The eight original research articles compiled in this thematic collection deliver multi-site, multi-mechanistic empirical evidence spanning Northeast China mollisols, Shanxi dryland spring maize systems, controlled hydroponic seedling assays, and molecular transcription factor functional dissection, collectively advancing our systematic understanding of how to remodel maize source–sink relationships, stem mechanical strength, root physiological vitality, and stress resilience for optimized production.

A core bottleneck restricting dense-planting maize yield stability is the trade-off between elevated population photosynthetic capacity and aggravated lodging risk, a challenge comprehensively addressed in the high-latitude maize belt of Northeast China [1,2]. Both studies converge on a synergistic management paradigm: moderate nitrogen supply (240 kg N ha^−1^) combined with chemical plant growth regulation (diethyl aminoethyl hexanoate · ethephon). Under a uniform high density of 90,000 plants ha^−1^, this integrated strategy exerts dual beneficial modulation [1]. On the source side, it elevates leaf SPAD value (a dimensionless relative index measured by a chlorophyll meter, which is widely used to estimate leaf chlorophyll content non-destructively), net photosynthetic rate (P_n_), and the activities of key C_4_ photosynthetic carboxylases RuBPCase and PEPCase, while optimizing canopy leaf area index and light interception distribution. On the sink–support side, chemical regulators shorten plant height and lower the gravity center, thicken basal internodes, and boost stem lignin, cellulose, and hemicellulose accumulation via upregulated nitrogen metabolism enzymes (GS, GDH, GPT). Beyond yield promotion, research further expands the environmental dimension of this technology, verifying that coordinated nitrogen–PGR management stimulates root morphological development and grain-filling enzyme activity to raise kernel weight, while cutting greenhouse gas fluxes relative to excessive nitrogen input [2]. Contrastingly, over-application of nitrogen (360 kg N ha^−1^) breaks this balance, elongating basal internodes, weakening stalk mechanical strength, and triggering severe lodging and nitrogen waste—an instructive warning against blind high-nitrogen fertilization under dense planting.

Population configuration, encompassing planting density and row layout, fundamentally shapes intraspecific and interspecific crop competition and resource partitioning. Research establishes an optimal density–nitrogen match (75,000 plants ha^−1^ + 180 kg N ha^−1^) for Shanxi dryland spring maize [3]. Rising planting density independently deactivates lignin-synthesis enzymes (PAL, TAL, CAD) and deteriorates stalk mechanical traits, yet rational nitrogen supplementation reverses this suppression; significant positive correlations between stalk breaking resistance and the three phenylpropane pathway enzymes provide a biochemical marker system for lodging-resistant cultivar screening and nitrogen regime calibration. For diversified cropping systems, research explores maize–alfalfa intercropping, a vital model balancing grain and forage output in Northeast China [4]. Among six row ratio treatments, the 2:2 maize–alfalfa configuration achieves the most balanced interspecific competition complementarity, maximizes land equivalent ratio (LER), system productivity index (SPI), and total intercropping yield, outperforming sole cropping by over 55%. In contrast, layouts with excessive maize rows strengthen asymmetric competitive dominance of maize over alfalfa and reduce overall system resource-use efficiency. Together, these two works highlight that population optimization cannot be separated from matched nutrient management—fine-tuning spatial crop distribution alone fails to unlock full yield potential without coordinated nitrogen input regulation.

Conservation tillage and soil organic matter amendment represent another pivotal pathway to upgrade maize productivity via root–shoot physiological interaction, focusing on thin-layer mollisols of the Songnen Plain [5]. Long-term intensive tillage degrades soil structure and suppresses root development, limiting post-anthesis dry matter and nitrogen translocation to grains. Deep straw incorporation (DF) outperforms surface straw mulching and conventional plowing by dramatically improving root architecture (length, surface area, volume) and root bleeding sap physiological activity. Enhanced root nutrient transport capacity further stimulates leaf photosynthetic enzyme activity and nitrogen assimilation efficiency, ultimately elevating 100-kernel weight and final grain yield by 8.34%. Structural equation modeling (PLS-SEM) quantitatively clarifies the causal chain: straw return remodels root morphology and bleeding physiology, which sequentially enhances canopy photosynthesis, post-flowering biomass accumulation, and nitrogen partitioning toward kernels. This mechanistic framework solidifies the theoretical foundation for straw return as a root-centered yield-improving practice in black soil regions.

Beyond field agronomic regulation, this collection expands research boundaries to abiotic toxic stress, drought tolerance biostimulants, and molecular genetic dissection of yield determinants, forming a multi-scale research spectrum from gene to farmland. Research reveals a biphasic dose effect of veterinary antibiotics (oxytetracycline, ciprofloxacin) on maize seedlings under hydroponic culture: low concentrations (3–5 mg L^−1^) boost photosynthetic enzyme activity, nitrogen metabolism enzyme levels, growth-promoting endogenous hormones (IAA, GA, ZR), and root vitality, whereas high residual concentrations (30–120 mg L^−1^) induce severe physiological inhibition, cell membrane damage, and disrupted hormone homeostasis [6]. This work alerts researchers of agricultural ecosystems to the hidden risks of antibiotic pollution from livestock manure, providing concentration thresholds for ecological risk assessment. For drought stress mitigation, research validates the combined application of zinc sulfate and plant growth-promoting Pseudomonas spp. as a sustainable drought tolerance strategy [7]. Seed priming emerges as the most efficient application mode, enhancing antioxidant enzyme activity, osmotic adjustment substance accumulation, and chlorophyll retention to offset drought-induced yield loss, offering a low-cost, microbe-based agronomic tool for water-limited maize regions. At the molecular regulatory level, research conducts a genome-wide identification of the maize HD-ZIP transcription factor family and functionally verifies ZmHD-ZIP23 as a positive regulator of seed size [8]. Overexpression of this gene enlarges embryo and endosperm volume, raising grain length and hundred-kernel weight. This discovery identifies a valuable gene target for molecular breeding of high-yield maize varieties, bridging the gap between basic transcriptional regulation research and applied yield improvement.

Collectively, the eight studies in this thematic issue construct an integrated research logic spanning molecular physiology, seedling toxicology, rhizosphere soil regulation, crop population configuration, integrated nitrogen–chemical regulation, intercropping systems, and environmental footprint evaluation. They share a consistent core objective: breaking the antagonistic relationships between high planting density and lodging risk, high yield and nitrogen waste, single-crop productivity and interspecific competition, intensive cultivation and soil degradation, and crop growth and abiotic stress damage. Several research gaps and forward-looking directions emerge from this body of work to guide future maize agronomy research. First, multi-site cross-region validation is required for integrated nitrogen–PGR dense planting technology, to disentangle genotype × environment × management interactions across distinct soil and climate zones. Second, the molecular signaling pathways linking root physiological status, leaf photosynthesis, and stem lignification under straw return and intercropping systems remain insufficiently characterized; transcriptomic and metabolomic profiling could uncover upstream regulatory networks. Third, antibiotic residual stress in actual farmland soil requires long-term field monitoring beyond short-term hydroponic seedling tests to quantify cumulative impacts on whole-growing-season maize performance. Fourth, synergistic coupling of microbial inoculants, zinc fertilizer, and PGRs deserves further exploration to develop multi-functional, low-input green cultivation regimes. Finally, translational research connecting transcription factor gene editing with field agronomic management must be strengthened to accelerate the deployment of high-yield, stress-resilient maize germplasm in production.

Maize production systems face compound pressures of food security demand and ecological protection. This collection demonstrates that coordinated physiological and agronomic regulation—rather than single-factor input increase—is the sustainable path forward to achieve synergistic gains in yield, lodging resistance, nutrient-use efficiency, and environmental friendliness. We anticipate that these findings will inspire follow-up research in maize physiology, cropping system optimization, conservation tillage, stress mitigation, and molecular breeding, and provide actionable technical references for smallholder farmers and large-scale commercial maize production across China and analogous global maize-growing regions.

We appreciate all anonymous reviewers for their detailed comments and valuable revision suggestions that greatly improved the scientific rigor and readability of each article. Special thanks go to the editorial team of *Plants* for efficient coordination throughout the whole collection, review and publication process. We hope the research outputs collected in this Special Issue can inspire further breakthroughs in maize physiological ecology and accelerate the transformation of efficient and stable maize cultivation technologies globally.

## Data Availability

No new data were created or analyzed in this study. Data sharing is not applicable to this article.
